# Bacteriophage Administration Reduces the Concentration of *Listeria monocytogenes* in the Gastrointestinal Tract and Its Translocation to Spleen and Liver in Experimentally Infected Mice

**DOI:** 10.1155/2010/624234

**Published:** 2010-06-24

**Authors:** Volker Mai, Maria Ukhanova, Lee Visone, Tamar Abuladze, Alexander Sulakvelidze

**Affiliations:** ^1^Department of Microbiology and Cell Science, University of Florida, Gainesville, FL 32611, USA; ^2^Emerging Pathogens Institute, University of Florida, Gainesville, FL 32610, USA; ^3^Intralytix, Inc., Baltimore, MD 21202, USA

## Abstract

To investigate the efficacy of phage supplementation in reducing pathogen numbers, mice were treated via oral gavage with a *Listeria monocytogenes* phage preparation (designated ListShield) before being orally infected with *L. monocytogenes*. The concentrations of *L. monocytogenes* in the liver, spleen, and intestines were significantly lower (*P* < .05) in the phage-treated than in the control mice. Phage and antibiotic treatments were similarly effective in reducing the levels of *L. monocytogenes* in the internal organs of the infected mice. However, the significant weight loss detected in the control and antibiotic-treated groups was not observed in the infected, ListShield-treated mice. Long-term (90 days), biweekly treatment of uninfected mice with ListShield did not elicit detectable changes in the microbiota of their large intestines or deleterious changes in their health. Our data support the potential feasibility of using bacteriophages to control proliferation of *L. monocytogenes* in mice without affecting commensal microbiota composition.

## 1. Introduction

Food borne bacterial pathogens remain a major health threat. Beyond reducing pathogen load at the source and in the final product, few measures are currently available to protect the human host. Side effects associated with long-term antibiotic treatment and the danger of emerging novel antibiotic resistance strains make an antibiotic-based prevention regimen unfeasible. In contrast, promising efforts are now being directed towards utilizing our own commensal microbiota to improve resistance to pathogens. The conventional approach aims at reshaping microbiota composition towards beneficial bacteria by adding live probiotic bacteria and/or by enhancing their growth through addition of prebiotic supplements [1]. An alternative approach for shaping overall microbiota composition aimed at reducing detrimental and potentially pathogenic bacteria by means of specific bacteriophages has to date received much less attention.

The human gastrointestinal (GI) tract is colonized by an abundant and diverse microbiota that plays a significant role in mucosal protection, regulation of GI immune tolerance, digestion of complex macromolecules including mucus and fiber, and vitamin K synthesis [2, 3]. Numerous factors (e.g., age, antibiotic treatment, diet, psychological and physical stress, hormone levels, etc.) may lead to physiological disturbances in the gut's microbiota [4]. Such alterations may contribute to many chronic and degenerative diseases, including Crohn's disease, ulcerative colitis, rheumatoid arthritis, irritable bowel syndrome, inflammatory bowel disease, and other “intestinal dysbioses” [5, 6]. Modification of gut microbiota by pre- and probiotics has been shown to modify risk for a variety of diseases, modulate of cell proliferation, and decrease serum cholesterol [7, 8].

Bacteriophages (phages) are viruses that attach to their specific bacterial hosts and kill them by sequential internal replication and lysis. Phages are the most ubiquitous organisms on Earth (their total number has been estimated to be 10^30^ to 10^32^), and they are believed to play a key role in establishing microbial balance in every ecosystem in which they are present [9]. Phages are abundant in saltwater, freshwater, soil, plants, and animals, and they frequently have been isolated from drinking water [10–17], and from a wide range of food products, including ground beef, pork sausage, chicken, farmed freshwater fish, common carp and marine fish, oil sardines, raw skim milk, and cheese [18–23]. In humans, prodigious numbers of phages “colonize” the GI tract, and they have been isolated from the skin, mouth, vagina, urine, and feces [24, 25] (for a review, see [26]). 

The use of phages in humans is very safe, as summarized in numerous recent review articles [26–34]. Thus, we hypothesized that phage administration might be safe and effective means for reducing or eliminating food borne and waterborne bacterial pathogens in the gut without altering the normal gut's microbiota; thereby, eliciting significantly fewer deleterious side effects compared to antibiotics. If our hypothesis is proven to have merit, regular administration of bacteriophages (e.g., similar to a probiotic preparation which is part of the daily diet) may in the future provide a natural, safe, and gentle means for maintaining healthy gut microbiota and protecting against specific food borne and waterborne pathogens. The current report presents the results of our proof-of-concept, *in vivo* studies examining a *L. monocytogenes* specific bacteriophage preparation's effect on the (i) concentrations of *L. monocytogenes* in internal organs and associated health effects in experimentally-infected mice, and (ii) overall health of mice including normal gut microbiota composition after long term exposure to our phage preparation.

## 2. Materials and Methods

### 2.1. Phage Preparation

ListShield (formerly LMP-102), a *L. monocytogenes*-specific phage preparation developed by Intralytix, Inc., was used during our studies as the prototype phage-based probiotic preparation. ListShield is a mixture/cocktail of six naturally occurring bacteriophages with strong lytic potency against *L. monocytogenes*. ListShield has been approved by the Food and Drug Administration as a *L. monocytogenes*-specific food additive for ready-to-eat meats (21 CFR §172.785), and by the Environmental Protection Agency as an environmental decontaminant for use in various food processing plants and establishments (EPA registration no. 74234-1). ListShield lots no. 0108B070117 and 0108D160161 were used during our studies.

### 2.2. Bacterial Strains

*L. monocytogenes* strain *Lm*370 was used to experimentally infect mice. It is a nalidixic acid-resistant mutant of ATCC strain 49594 derived from the Scott A strain. The mutant was selected by serially passaging ATCC strain 49594 on MOX agar plates supplemented with increasing concentrations of nalidixic acid. The strain underwent ≤8 serial passages before it was determined to be nalidixic acid-resistant at a concentration of ca. 50 ng/ml. *Lm*370 also is sensitive to ampicillin and ListShield. Its sensitivity to ampicillin was determined by a standard disk diffusion method, and its sensitivity to ListShield was determined as described below. The strain was stored frozen (−80°C) in 30% glycerol/70% LB broth, plated on MOX agar prior to use, and grown in LB broth (30°C, overnight) before being administered to mice by oral gavages. The MOX agar and LB broth used to grow the bacterium were supplemented with nalidixic acid (50 ng/ml). The following 21 non-*L. monocytogenes* strains were used to confirm the in vitro specificity of ListShield: two *Enterococcus faecalis *strains (1) ATCC 11823, and (2) ATCC 19433; eight *E. coli* strains (1) ATCC 700728, (2) ATCC 35321, (3) ATCC 35322, (4) ATCC 35335, (5) ATCC 35342, (6) ATCC 35343, (7) ATCC 35351, (8) ATCC 35352; one *Shigella sonnei* strain ATCC 9290; four *Pseudomonas aeruginosa* strains (1) ATCC 15692, (2) ATCC 51674, (3) ATCC 43390, (4) ATCC 39324; and six *Salmonella enterica* subsp. *enterica* strains, including (1-2) serovar Typhimurium strains ATCC 19585 and ATCC 13311, (3) serovar Gallinarum strain ATCC 9184, (4) serovar Newport strain ATCC 6962, (5) serovar Enteritidis strain ATCC 13076, and (6) serovar Paratyphi B strain ATCC 10719.

### 2.3. Determining the In Vitro Specificity of Phage Preparation

The in vitro specificity of ListShield was confirmed by characterizing its ability to lyse 21 strains of the above-mentioned bacterial species other than *L. monocytogenes*. Lytic activity was detected with a classical spot-testing technique [35], by incubating bacterial lawns spotted with aliquots of diluted phage preparations (10^4^ PFU/ml) and examining the lawns for zones of lysis.

### 2.4. In Vivo Studies

Inbred C57BL/6J mice were obtained from Harlan (Harlan laboratories, Indianapolis, IN), acclimated to the laboratory environment for at least 3 days after arrival, and fed *ad libitum* with Harlan chow 7912 (Harlan laboratories, Indianapolis, IN) and water. The studies were conducted at the University of Florida (UFL), according to a protocol (no. FO97) approved by the UFL's Institutional Animal Care and Use Committee. The in vivo studies were performed as short-term (7 days) and long-term (90 days) experiments.

At the start of the short-term (7 days) study, each mouse was weighed and randomly assigned to three experimental groups: Group 1 (15 mice, the PBS control group) received phosphate-buffered saline (PBS, pH 7.4) daily for 3 days before and after challenge (ca. 10^5^ colony-forming units [CFU]) with strain *Lm*370; Group 2 (20 mice, the ListShield test group) was treated with ListShield (10^5^ PFU) daily for 3 days before and after challenge; and Group 3 (10 mice, the antibiotic test group) received PBS daily for 3 days prechallenge, and was treated with one dose of ampicillin (25 mg/g) 15 min postchallenge. All treatments and challenge doses of bacteria were administered by oral gavage in 0.1 ml PBS. After being weighed again, each mouse was sacrificed on day 7 by CO_2_ inhalation followed by cervical dislocation. After sacrifice, (i) the left lower lobe of the liver, (ii) the entire spleen, (iii) a 5-cm-long section of the small intestine, (iv) the cecum, and (v) fecal matter from the large intestine of each mouse were weighed and placed in separate aliquots (5 ml) of cold PBS and their concentrations of strain *Lm370 *were determined as described below. Also, fecal matter was frozen and subsequently analyzed to characterize microbiota profiles (see below). During the short-term study body weight was measured daily. 

For the long-term (90 days) study, each mouse was weighed and randomly assigned to two experimental groups (15 mice/group): Group 1 (test mice) received ListShield biweekly, in their drinking water, at a concentration of ca. 10^5^ PFU/ml; and Group 2 (control mice) received drinking water containing PBS biweekly. Five mice in each group were sacrificed by CO_2_ inhalation followed by cervical dislocation on days 30, 60, and 90 after their first ingestion of ListShield. Final body weight was measured before liver, spleen, and small intestinal tissues were removed and examined for histopathological changes. Also, specimens of unclotted blood and fecal matter were obtained on day 90 from each mouse. The former were analyzed for their concentrations of various white blood cells (WBC), and the latter were frozen and subsequently analyzed to characterize microbiota profiles (see below). 

### 2.5. L. monocytogenes Enumeration and Phage Titers

Tissues of internal organs and fecal matter were ground in Dulbecco's PBS (pH 7.0) with a tissue homogenizer, and aliquots (0.1 ml) of each preparation were spread, in duplicate, on MOX agar plates supplemented with nalidixic acid (50 ng/ml). The plates were incubated (30°C, 48 h), the number of viable *L. monocytogenes* was estimated by standard colony counting, and the concentrations were expressed as the number of CFU/g of specimen. Concentrations of ListShield phages in the samples were determined by filtering the samples through 0.45 *μ*m filters and by analyzing the filtrates using standard plague counting assay [36].

### 2.6. Enumeration of WBC

The concentrations of granulocytes (neutrophils, basophils, and eosinophils) and agranulocytes (lymphocytes and monocytes) were determined in blood samples obtained from mice at the end of the long-term study. The analysis was performed in the animal facility under supervision of a veterinarian pathologist.

### 2.7. Microbiota Denaturing Gradient Gel Electrophoresis (DGGE) Profiles

Bacterial genomic DNA was isolated from the large intestinal contents as previously described [37]. A 457-bp fragment from the V6 to V8 region of the bacterial 16S rDNA gene was amplified with primers U968-GC (5′ CGC CCG GGG CGC GCC CCG GGC GGG GCG GGG GCA CGG GGG GAA CGC GAA GAA CCT TAC) and L1401 (5′GCG TGT GTA CAA GAC CC), as described by Zoetendal et al. [38]. DGGE was performed in an 8% (wt/vol) polyacrylamide gel with a denaturing gradient ranging from 40% to 50% at the top and bottom of the gel, respectively (100% denaturing conditions were defined as 7 M urea and 40% formamide). After electrophoresis (16 h, 65 V, 60°C), the gels were stained with SYBER Green (Novex, San Diego, CA) and scanned/analyzed with Quantity One and Diversity Database software (Bio-Rad, Hercules, CA).

### 2.8. Statistical Analysis

The significance of differences in the *L. monocytogenes* concentrations in the test and control groups of mice was determined in MS Exel using two-tailed *t*-tests. The relatedness of the microbiota profiles was calculated using Pearson correlation coefficients and the gel analysis software package Diversity Database (Bio-Rad, Hercules, CA). Microbiota diversity was determined using the Shanon Wiener index and the Simpson diversity index.

## 3. Results

An animal model closely resembling human listeriosis is currently not available. Thus, we used a simplified model in which mice were challenged by oral gavage with a single dose of *L. monocytogenes* strain *Lm*370. *L. monocytogenes* persisted in the animals for at least 3 days and in all untreated mice translocated to spleen and liver. Out of 45 mice two mice in the PBS-treated group, two mice in the ampicillin-treated group, and one mouse in the ListShield-treated group died prematurely, likely from stomach puncture during oral gavage. These mice were excluded from the analysis. Thus, data from 13 mice in the PBS-treated/control group, 8 mice in the ampicillin-treated test group, and 19 mice in the ListShield-treated test group were retained in the short-term study.

**Figure 1:**
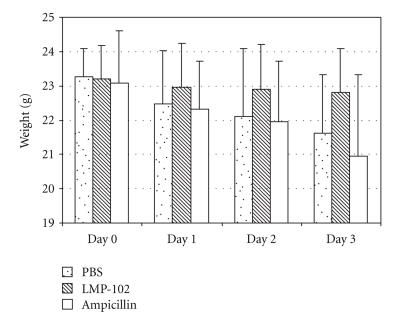
Mean body weights of mice infected with *L. monocytogenes* and treated with PBS, ListShield, and ampicillin. The results shown are for the days postchallenge with strain *Lm*370. The standard deviations are indicated with brackets.

Body weight did not differ between the groups at the time of randomization. Within three days of challenge with *L. monocytogenes* mice in the PBS treated and in the ampicillin treated groups lost up to 10% of their body weight (Figure 1). In contrast, mice in the ListShield treated group maintained their body weight; the difference in weight loss between these groups was statistically significant (*P* < .05). The body weight loss in mice treated with PBS and ampicillin was due to diarrhea that was observed shortly after bacterial challenge and at necropsy. Thus, we found that treatment with our *L. monocytogenes*-specific phage preparation (ListShield), but not with PBS or ampicillin, significantly reduced diarrhea associated weight loss.

**Figure 2:**
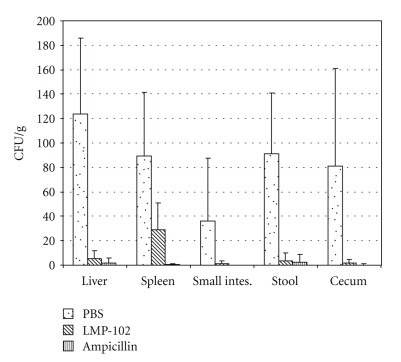
Recovery of *L. monocytogenes* from experimentally-infected mice treated with PBS, ListShield, and ampicillin. The bacterial concentrations are expressed as the number of CFU/g of specimen. The results shown are for 3 days postchallenge with strain *Lm*370. The standard deviations are indicated with brackets.

All of the challenged mice were sacrificed on day 7 (4 days postchallenge) and the concentrations of strain *Lm*370 were determined in their livers, spleens, and intestines. The levels of *Lm*370 in all of the examined specimens were significantly lower (*P* < .05) in the ListShield- and ampicillin-treated groups than in the PBS treated mice (Figure 2). The *Lm*370 concentrations in tissues from the ampicillin- and ListShield-treated mice were similar (difference not significant; *P* > .05), indicating that both treatments were effective in reducing *L. monocytogenes* colonization/infection in this mouse model. We monitored persistence of bacteriophage in fecal pellets and cecal contents. However, phage titers were below detection limits of the assay suggesting that phage amplification in the gut environment was limited. 

At necropsy, we observed that the mice treated with ampicillin, but not those treated with ListShield, exhibited watery large intestinal contents and enlarged ceca. This observation suggests that the broad-spectrum antibiotic (ampicillin), while reducing the *L. monocytogenes* levels, deleteriously affected normal gut microbiota, which resulted in the diarrhea and weight loss we observed. Because one dose of ampicillin was sufficient to remove most challenge bacteria, we only administered the antibiotic once after challenge. Importantly, the detrimental effects of ampicillin treatment on the GI microbiota of mice were still more obvious than in the mice receiving three postchallenge doses of ListShield. In order to further evaluate this possibility, we used 16S rRNA-based DGGE to characterize and compare the microbiota profiles in the intestinal contents of the three groups. The intensity of some DGGE bands was increased in the ampicillin-treated group, which suggests that ampicillin-treatment caused overgrowth of some of the normal intestinal bacteria. However, this suggestive difference in overall microbiota diversity was not statistically significant when determined using Shannon Wiener and Simpson diversity indexes (*P* > .05). 

**Figure 3:**
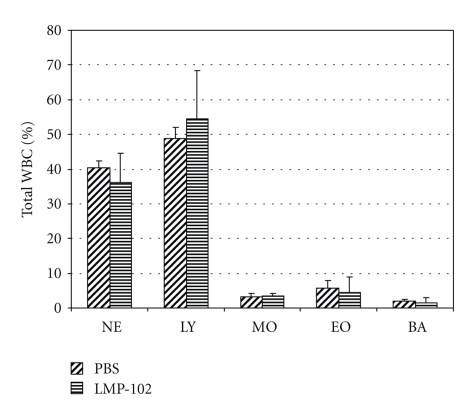
Concentrations of various white blood cells (WBC) in uninfected mice after long-term (90 days) ingestion of ListShield. The standard deviations are indicated with brackets. NE: neutrophils; LY: lymphocytes; MO: monocytes; EO: eosinophils; BA: basophils.

To determine potential side effects of long-term phage administration, we performed a 90-day-long study of ListShield (supplied via drinking water) on overall health and large intestinal microbiota of mice not infected with *L. monocytogenes*. On days 0, 30, 60, and 90 of the long-term study, the mean body weight of the mice comprising the ListShield test group was not significantly different from that of the mice in the PBS control group. After 90 days, the white blood cell concentrations in the ListShield test mice were not significantly different from those of the PBS control mice (Figure 3). Also, blood chemistry and tissue pathology did not show gross abnormalities (Table 1). We did detect a minor increase in lymphocyte infiltrate into the duodenum and mild dermatitis in the LMP-102 group. These findings indicate little changes in overall health. 

**Figure 4:**
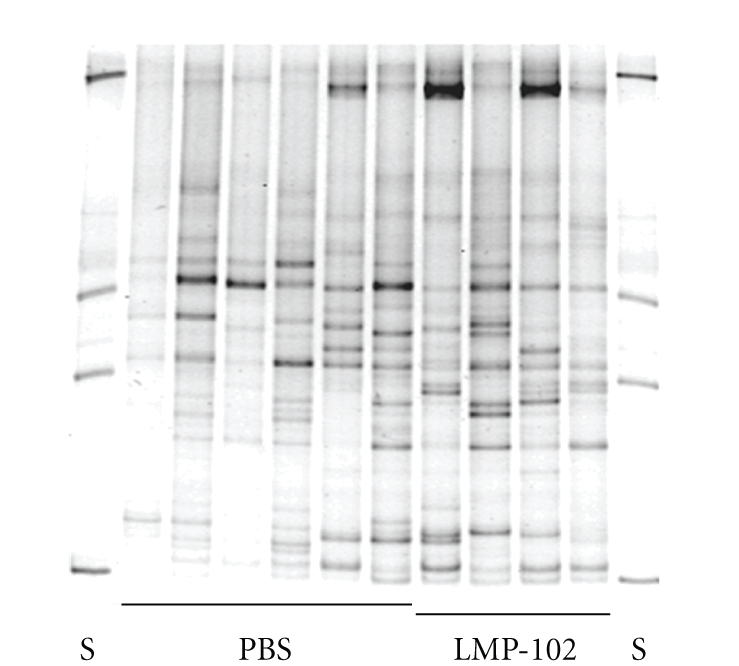
DGGE profiles of the microbiota in fecal specimens obtained from uninfected mice after long-term (90 days) ingestion of ListShield. S: standards (DNA from mix of 16S rRNA clones); PBS: profiles of the fecal microbiota in PBS control mice; LMP-102: profiles of the fecal microbiota in ListShield test mice.

The lytic activity of ListShield was limited to the targeted *L. monocytogenes* strains, and the preparation did not lyse in vitro any of the 21 strains of other bacterial species we examined. Our in vivo data were in agreement with these observations: oral administration of ListShield did not alter the normal diversity of the GI tract microbiota. Although the DGGE analysis did reveal some differences between the DGGE band profiles of the fecal microbiota in the ListShield group; overall diversity in the PBS control mice was similar (Figure 4).

## 4. Discussion

The rationale for our studies was based on the hypothesis that regular, long-term ingestion of bacteriophages (e.g., similar to a probiotic preparation consumed as part of the daily diet) can be safely used to significantly reduce the levels of specific bacterial pathogens in the mammalian gastrointestinal (GI) tract and reduce translocation to internal organs. The key difference between the conventional approach used for bacteria-based probiotics and our current approach for phage-based preparations is that the former introduces nonpathogenic bacteria into the GI tract in order to improve immune balance colonization resistance; whereas, the latter directly removes specific pathogenic bacteria from the GI tract. 

The animal model used during our studies does not closely mimic human listeriosis; therefore, extrapolations about the potential comparable efficacy of ListShield and ampicillin in preventing and treating human disease cannot be made at this time. ListShield was administered at a high dose soon after infection with Listeria. Also, numbers of Listeria in the GI tract of a human host would be low making maintenance of an infectious cycle difficult for phage propagation. We observed a large variation in pathogen load among individual mice possibly masking a statistically significant increase in *L. monocytogenes* in spleen tissue in ListShield group compared to the ampicillin group (Figure 2). Nevertheless, our data do (i) suggest that oral administration of ListShield can significantly reduce the number of viable *L. monocytogenes* in the GI tract and the reticuloendothelial system (e.g., the liver and spleen) of orally infected mice, and (ii) support our general hypothesis that probiotic-type phage administration is capable of significantly reducing in vivo colonization by their pathogenic bacterial hosts.

The observation that ListShield did not lyse any of the non-*L. monocytogenes* strains was expected given the known high specificity of bacteriophages towards their host strains. Our data provided further support to this idea. In the same context, our microbiota analysis did not reveal significant differences between the DGGE profiles of the fecal microbiota in the ListShield test mice and the PBS control mice. Although the sensitivity of DGGE was not optimal the data we obtained during our in vitro sensitivity examination of ListShield, and the short-term and long-term in vivo studies, suggest that oral ingestion of ListShield affects the overall composition of the large intestine's microbiota less than does ampicillin ingestion. Overall, our results suggest that (i) using bacteriophages may be a similarly effective but gentler approach than using antibiotics for reducing *L. monocytogenes* colonization/translocation, and (ii) ListShield can be administered to mice without any detectable side effects over a relatively long period of time (at least 90 days). Our approach of using phages as probiotics for specifically targeting bacterial pathogens in the GI tract may have some important public health implications, and it may provide an effective and gentle means for preventing or reducing the severity of disease caused by *L. monocytogenes* and, potentially, other foodborne or waterborne pathogens that have an oral portal of entry and require short- or long-term colonization of the GI tract in order to cause disease.

## Figures and Tables

**Table 1:** Tissue histology and blood chemistry in the two groups (N = 5 mice/group), N = normal, I = infiltrate, D = dermatitis.

	Kidney	Liver	Spleen	Lung	Heart	Stomach	Duodenum	Skin	Blood
PBS	N	N	N	N	N	N	N	N	N
LMP-102	N	N	N	N	N	N	I	D	N
